# Insight into *Bacillus cereus* Associated with Infant Foods in Beijing

**DOI:** 10.3390/foods11050719

**Published:** 2022-02-28

**Authors:** Ping Wang, Xiaomei Zhao, Tianming Qu, Lijiao Liang, Qinglong Ji, Ying Chen

**Affiliations:** Chinese Academy of Inspection and Quarantine, Beijing 100176, China; wangp_129@163.com (P.W.); xiaomei200413@126.com (X.Z.); qtm110506@163.com (T.Q.); lianglijiao96@163.com (L.L.); jql81070305@aliyun.com (Q.J.)

**Keywords:** infant formula, *Bacillus cereus*, virulence genes, antimicrobial drugs susceptibility

## Abstract

This study was undertaken to investigate the prevalence, antimicrobial resistance, and virulence gene profiles of *Bacillus cereus* in different brands of infant formula in Beijing supermarkets. Eighty-eight *Bacillus cereus* isolates were recovered in sixty-eight infant formulas of five domestic brands and fourteen imported brands. The prevalence rate in domestic and imported samples were 70.6% and 52.9%, respectively. Lower mean prevalence level was found in domestic samples (1.17 MPN/g) compared with the imported samples (3.52 MPN/g). Twenty-four virulence gene profiles were found, and most strains carried at least one virulence gene. The prevalence of *nheA*, *nheB*, *nheC*, *cytK*, *bceT,* and *entFM* in domestic and imported brand samples was similar. The occurrence of enterotoxin genes *hblA*, *hblC,* and *hblD* in domestic samples were 22.2%, 27.8%, and 22.2%, respectively, which was significantly higher than imported samples. Antimicrobial drugs-susceptibility analysis showed that all isolates were susceptible to gentamincin, amikacin, and ciprofloxacin; 38%, 7%, and 2.3% were resistant to rifampin, tetracycline, and chloramphenicol, respectively; and only one isolate was resistant to trimethoprim-sulfamethoxazole. Moreover, the cell numbers of *Bacillus cereus* in prepared infant formula increased rapidly at room temperature. Thus, monitoring guidelines are needed for accepted levels of *Bacillus cereus* in infant formula.

## 1. Introduction

Infant formula is a major source of nutrition for children before they can digest other foods. The immune system of babies is weak, and any pathogen present in their food may cause illness. Therefore, the hygienic quality of infant formula is important to protect the health of infants and to diminish risks and associated with its consumption. As one of the high-protein foods, dairy products are conducive to foodborne pathogen proliferation. A growing research has suggested that *Bacillus cereus* is a common pathogen in milk or milk-related products [1,2].

*Bacillus cereus*, a spore-forming bacterium, is an opportunistic human food-borne pathogen, which is widely distributed in environment and frequently isolated as a contaminant of cereals, processed milk products, and other foods [3,4]. The spores of *B. cereus* are specifically troublesome in the food industry because they can be intractable to pasteurization, radiation, disinfectants, and desiccation, and their hydrophobic nature allows them to adhere to the surface of solid materials [5,6]. In addition to gastroenteritis, *B. cereus* can also cause systemic and local infection in immunologically compromised individuals [7].

*Bacillus cereus* is capable of producing emesis toxin (ETE) and three enterotoxins, including hemolytic enterotoxin (HBL), nonhemolytic enterotoxin (NHE), and enterotoxin K (EntK), of which HBL and NHE are important enterotoxins that cause diarrhea-based food poisoning. There are five virulence genes associated with the production of these enterotoxins: hemolysin BL gene (*hbl*), non-hemolytic enterotoxin gene (*nhe*), enterotoxin FM gene (*entFM*), enterotoxin T gene (*becT*), and cytotoxin K gene (*cytK*). HBL requires all three genes positive for *hblA*, *hblC*, and *hblD* to be toxic, while NHE is most toxic when all three genes are positive for *nheA*, *nheB*, and *nheC*. The virulence gene expressing vomitoxin is *ces* gene, which encodes a heat-resistant toxin that is not easily cleaved [4,8]. A total of 24 virulence gene carriage patterns of *B. cereus* were identified, among which the *nhe* gene had the highest carriage rate of 92.98%, followed by the *entFM* gene (71.93%), and 70.18% of strains carried both *nhe* and *entFM* genes. The subtyping results showed that the carriage rates of *nheA*, *nheB,* and *nheC* genes were 88.72%, 88.72%, and 49.12%, respectively. The hemolysin BL gene carriage rates were 24.56% for *hblA*, 22.81% for *hblC,* 17.54% for *hblD*, and 22.81% for *cytK* [8,9].

Besides the enterotoxic toxins, it has been found that some *B. cereus* isolates show resistance to antimicrobial drugs [10]. A variety of antimicrobial drugs are widely used, and the problem of bacterial resistance is getting worse, causing great threat to human health. With antimicrobial agents widely used in farmed-animal industries, the food chain constitutes an important source of antimicrobial resistance [11]. There is evidence that resistant microorganisms can spread to humans via the food chain or indirect contact from farm animal waste [12,13]. Therefore, it is necessary to evaluate the antibiotic resistance of *B. cereus* in infant formula. 

Up to now, many countries have stipulated the limit standard of *B. cereus* in infant formula to control this bacterium. In Australia and New Zealand, it was (*n* = 5, *c* = 0, *m* = 100 cfu/g (mL)); in Canada, it was (*n* = 10, *c* = 1, *m* = 100 cfu/g (mL), *M* = 10, 000 cfu/g (mL)); and in the European Union, it was (*n* = 5, *c* = 1, *m* = 100 cfu/g (mL), *M* = 500 cfu/g (mL)). While in China, the national standard of infant formula, GB 10765-2010, does not stipulate the limit standard of *B. cereus* in infant formula, the control and inspection of *B. cereus* in infant formula is mainly according to a prevalence level less than 100, 000 cfu/g (mL). Therefore, it is important to determine the prevalence and also enumerate *B. cereus* in babies’ food in order to assess its safety. 

Therefore, the present study was undertaken to evaluate the prevalence and potential risk of *B. cereus* in infant formulas in Beijing. The research was further extended to carry out the virulence genes profile, and antimicrobial-resistance profiles of the isolates were evaluated.

## 2. Materials and Methods

### 2.1. Sample Collection

A total of 68 infant formula samples were collected from supermarkets in Beijing during the period from June to July 2014. Among the 68 samples, 17 were from different domestic brands, and the other 51 samples involved 14 different imported brands. The samples of every brand were from different batches (1 to 5). The infant formulas selected in this study included most of the popular brands in the Chinese retail market.

### 2.2. Microbiological Analysis

The *B. cereus* in infant formula was detected by using a most probable number (MPN) procedure method described by Tallent et al. [14]. Briefly, 25 g of each sample was suspended in 225 mL of 0.85% saline and beaten for 1 min by a homogenizer (BAGMIXER100, interscience, France). Then, 10 mL of homogenate was serially diluted (10-fold) in 0.85% physiological saline with selected 3 continuous dilution, and then, 3 × 10 mL of each dilution was inoculated into 3 tubes with 10 mL double trypticase soy polymyxin broth (TSPB, Difco, 7 Loveton Circle,Sparks, MD, USA), followed by incubation at 30 °C for 48 h. The culture was then taken from tubes streak and applied onto mannitol yolk polymyxin (MYP) agar (Difco, 7 Loveton Circle, Sparks, MD, USA) and then incubated at 30 °C for 24 h. The typical colonies from plates that contain suspicious colonies were cultured on blood plates (Oxoid, Hampshire, UK) incubated at 30 °C for 24 h. The colony on the blood plates were confirmed by BD PhoenixTM-100 (Becton, Dickinson and Company, 7 Loveton Circle Sparks, USA), and the *gyrB* gene was detected using a previously described PCR method for the identification of *Bacillus cereus* [15]. The strains were conserved in Inspection and Quarantine of China microbial culture collection management center (IQCC).

### 2.3. Detection of Virulence Genes

The strains isolated from infant formula samples were screened by PCR for the ten virulence genes, including *hblA*/*C*/*D*, *nheA*/*B*/*C*, *cytK*, *bceT*, *ces,* and *entFM* [9]. For extraction of DNA template, bacteria were plated on Tryptone soya agar (TSA, Oxoid, Hampshire, England) and incubated at 30 °C for 24 h; then, 5 colonies were taken in 300 μL of Tris-EDTA buffer. The lysis of bacteria was performed by incubation at 100 °C for 10 min, and debris was removed by centrifugation at 15,000× *g* for 3 min. The DNA-containing supernatant was transferred to a new tube and stored at −20 °C. 

For amplification, Ex Taq kit (TaKaRa, Otsu City, Japan) was used, and 25 μL mixtures were as follows: 2.5 μL of 10×Ex Taq buffer, 0.25 μL Ex Taq DNA polymerase (5 U/μL), 2 μL dNTP mixture (2.5 mM), 2 μL template DNA, 0.5 μL of each primer (10 μM), and 17.25 μL ddH_2_O. The reactions were performed on a Veriti 96-Well Thermal Cycler (Applied Biosystems, Carlsbad, CA, USA) under the following conditions: 95 °C for 10 min; 35 cycles at 95 °C for 30 s, annealing temperature for 30 s, and 72 °C for 30 s; and a final extension at 72 °C for 10 min. The *B. cereus* ATCC 11,778, ATCC 33,019 strains were used as positive control, and *Salmonella typhimurium* IQCC 10,503 without virulence genes as detected in this study was used as negative control. PCR products were analyzed by electrophoresis in 2% agarose and stained with ethidium bromide (EB). Gel images were captured on a Versadoc Imager (Bio-Rad, Hercules, CA, USA).

### 2.4. Antimicrobial Drugs Susceptibility Test

Antimicrobial drugs susceptibility of the *B. cereus* strains was determined by means of the agar disk diffusion method as recommended by the Clinical Laboratory Standards Institute [16]. Nine antimicrobial drugs often used in clinical and farmed-animal industries area were evaluated, namely gentamicin (GM) (10 μg/disk), tetracycline (TET) (30 μg/disk), erythromycin (ERY) (15 μg/disk), chloramphenicol (CHL) (30 μg/disk), amikacin (AMK) (30 μg/disk), and ciprofloxacin (CIP) (5 μg/disk), clindamycin (CC) (2 μg/disk), trimethoprim-sulfamethoxazole (SXT) (1.25/23.75 μg/disk), and rifampin (RIF) (5 μg/disk). Inhibition zones were measured in millimeters and interpreted according to the CLSI instruction.

### 2.5. Simulated Survive Test

Two strains, both isolated from infant formula samples, were randomly selected and used to investigate whether formula supports *B. cereus* survival, and simulated samples were prepared. Briefly, two *B. cereus* isolates were respectively inoculated onto infant formula powder (25 g) at finial concentration at 10 MPN/g, and 225 mL DDW were added and then incubated at room temperature (22 °C) and 37 °C for 24 h. During incubation, simulated samples were tested every hour by the MPN procedure method described above.

### 2.6. Statistical Analysis

The rates of recovery of *B. cereus* among the domestic brand and the imported brand formula were compared using Student’s *t*-test. Student’s *t*-test was also performed to compare the prevalence of virulence genes.

## 3. Results

### 3.1. Prevalence of B. cereus in Infant Formula Samples

A total of 68 infant formula samples, including 51 imported brands samples and 17 domestic brands samples, were analyzed. Among 51 imported brands samples, 27 samples were positive for *B. cereus,* with the prevalence rate of 52.9% and the mean prevalence level of 3.52 MPN/g (Table 1), while in 17 domestic brands samples, 12 samples were positive, with prevalence rate of 70.6% and the mean prevalence level 1.17 MPN/g. Among the samples positive for *B. cereus*, thirty-three had prevalence level < 3 MPN/g, four of them measured between 3~10 MPN/g, and only two of samples were measured to a level of 11~50 MPN/g. Despite the prevalence rate of *B. cereus* in domestic brands, the sample was higher than the imported brands sample; however, the difference was statistically not significant at a 5% level.

### 3.2. Virulence Genes Profiles of B. cereus

Eighty-eight isolates were identified as *B. cereus*, the multiplex PCR was carried to detect the virulence genes, and most isolates carried *nheC* gene, followed by *nheB* and *nheA,* which were 84.1%, 63.6%, and 54.5%, respectively. The occurrence of three genes of hemolytic enterotoxin complex (HBL) of *hblA*, *hblC,* and *hblD* were 5.68%, 9.09%, and 5.68%, respectively. The frequency of *cytK* gene was 21.6%. The *ces* gene encoding emetic toxin was only found in three (3.41%) isolates (Table 2).

About 24 virulence gene profiles of *B. cereus* were detected (Table 3), and the predominant profile was XII (22.73%), in which NHE complex genes were positive and the other virulence genes were negative by PCR test. In profile XII, seventeen strains were isolated from imported formula samples. Additionally, there were 53.4% (47/88) *B. cereus* isolates with *nheA*, *nheB,* and *nheC* simultaneously, while 4.55% (4/88) strains carried three *hbl* genes. Three isolates include all three types of both *nhe* and *hbl*. However, thirteen strains were without any of the virulence genes. Statistical analysis suggested the prevalence of *nheA*, *nheB*, *nheC*, *cytK*, *bceT,* and *entFM* in domestic brand samples were similar with imported samples (*p* > 0.05), ranging from 18.6% to 64.3%, while *hblA*, *hblC,* and *hblD* in domestic samples were 22.2%, 27.8%, and 22.2% significantly higher than in imported samples (*p* < 0.05).

### 3.3. Resistance to Antimicrobials

All of the 88 isolates were susceptible to GM, AMK, and CIP; seven strains were resistant to TET; two strains resistant against CHL; and only one isolate resistant against SXT. The strains had low sensitivity to RIF (Table 4). Furthermore, seven TET resistant isolates were also resistant to RIF. Seven strains with both TET resistance and RIF resistance were detected in this study, and no multiple antibiotic-resistant isolates were detected.

### 3.4. Simulated Survive Test

To investigate whether prepared infant formula supports *B. cereus* growth, two strains were randomly selected for the simulated survive test. *B. cereus* MPN levels were tested every hour for 24 h. At room temperature, the MPN reached 139/g and 127/g in two prepared simulated infant formula samples within ten hours. After 24 h incubation, *B. cereus* levels reached 1000 MPN/g, while increasing the temperature to 37 °C resulted in increased of *B. cereus* levels to 1000 MPN/g following only 6 h of incubation and 10,000 MPN/g after 24 h incubation.

## 4. Discussion

Foodborne diseases caused by foodborne pathogens have become one of the major safety issues that threaten human health. In particular, *Salmonella*, *E. coli*, *Staphylococcus aureus*, and *Listeria monocytogenes* are considered to be the most important and serious foodborne pathogenic bacteria [17]. *Bacillus cereus* is a neglected foodborne pathogen because of its strong environmental tolerance, spore-forming ability, and ability to produce toxins. It is often found in raw milk and dairy products.

In China, it is generally considered that imported brands of infant food are better than the domestic. However, the results of the present study proved that infant formulas were frequently found to include *B. cereus* although there was no significant difference between the imported and domestic infant formula samples (*p* > 0.05) for the prevalence of *B. cereus*. The result of the overall prevalence of *B. cereus* in infant formula was higher than a previously study [18], which reported that 14.08% of samples were contaminated with *B. cereus* from infant formula samples. The reason may be that *B. cereus* was examined by the plate count method in their study, while the MPN method was adopted in our study. The MPN method has lower detection limit than the plate count method and frequently was used when the level of *Bacillus cereus* ≤ 1000 CFU/g.

Infant formulas are prepared with warm water before consumption, and this organism may be activated by a normal process. If the temperature is adequate, one can assume that spores of *B. cereus* will be activated. The temperature of the water used to prepare the infant formula is advised to be 35 °C~60 °C, and this temperature may facilitate the activation of spores. Therefore, it is not surprising that some cases of *B. cereus* poisoning were linked to the infant formula [19,20]. Investigation was also conducted to study the possibility that large number of *B. cereus* could be ingested through the consumption of contaminated infant formula. Although most infant formula products in China have directions for use, it is possible that they may be consumed outside the instructions. Results of room temperature (22 °C) were examined to reflect situations such as those in some villages and towns or small childcare centers, where remaining infant formula food may not remain under refrigeration temperature. At room temperature, in simulated prepared infant formula samples, with an increase in cell number to an MPN of 100/g for 10 h after 24 h incubation, *B. cereus* numbers reached an MPN of 1000/g. However, increasing the temperature to 37 °C resulted in increases of *B. cereus* to an MPN of 1000/g after only 6 h of incubation and an MPN of 10,000/g after 24 h incubation. These data raise the concern that contaminated infant formula products available in China pose a potential risk to infants and raise the possibility that these products have already been a cause of illness in the past. Considering the prevalence level of *B. cereus* in foods that caused illness in the past, mostly ≥100,000 cfu/g, the level in determined in this study was quite low, indicating that the samples were relatively safe in terms of prevalence level at the time of purchase. However, the infant formula food can be safe only if it is consumed as per the guidelines given by the brands. In prepared infant formula that supports growth, the cell number is expected to increase at the time of consumption, especially when unfinished, prepared products are not properly maintained under refrigeration.

Virulence genes are thought to be linked with food spoilage, diarrhea, emesis, and other complications caused by *B. cereus* [21]. The diarrheal form of the syndrome has been associated mainly with hemolysin BL (*Hbl*), non-hemolytic enterotoxin (*Nhe*), and the cytotoxin K (*CytK*) [22,23]. For the detection virulence genes, we observed 75 isolates carried at least one virulence gene, and of them, *nhe* genes were the greatest (98.67%). This was consistent with what was reported in previous studies [9], which showed that *B. cereus* isolated from foods showed more frequent detection of NHE complex genes. HBL enterotoxin complex consists of B, L1, and L2, and its enterotoxic activity appears when all these components of the HBL complex are present [4,24]. Regarding the occurrence of the hemolytic BL genes, *hblCDA* among food strains isolated in Korea was 81.8% [24], while *hblC*, *hblD*, and *hblA* genes among *B.cereus* isolated in Chinese pasteurized, full-fat milk occurred with frequencies between 37.0% and 71.7% [25]. In this study, we found these three genes detection rates was 4.55%, lower than those mentioned above. Three isolates harbored all detected virulence genes; according to the labels, we found these strains isolated from two domestic-brand infant formulas. It is suspected that these isolates could be more virulent to humans, and the possibility of severe food poisoning case caused by these virulence genes might exist in China.

Foodborne pathogens that are resistant to a variety of antibiotics have become a major health concern [10]. Aminoglycosides, macrolides, and chloramphenicol antibiotics are usually recommended as the drugs of choice against *B. cereus* infections. In this study, some isolates were found to be resistant to macrolide and chloramphenicol antibiotics, which may be due to their identical action, acting on the 50S subunit of the bacterial ribonucleoprotein bodies and blocking protein synthesis. Seven multiple antibiotic isolates were also found, a result similar to previous studies, and *B. cereus* isolated from food was also found to be resistant to multiple antibiotics [9,11]. The isolation of these resistant *B.cereus* strains from infant formula is worrying. It is suggested that the inappropriate use of antibiotics in veterinary medicine contribute to a potential prevalence of raw materials of infant formula.

It is also found that strains isolated from one sample had different virulence gene profiles and antibiotic resistance. This might indicate the natural environment is reservoir for *B. cereus,* and food products are easily contaminated, and there is more than one contaminant source of infant formula. More isolated from infant formula, other foods and even some samples from the production environment are needed for subtyping and for infectious resource tracing and control.

## 5. Conclusions

The current findings suggest that the prevalence of *Bacillus cereus* in infant formula remains high and that antibiotic-resistance genes and virulence genes are present. Moreover, our data suggest that *B. cereus* may be an important pathogen of infant formula food poisoning in Beijing and needs to be controlled in some way. While in China, the regulation of *B. cereus* is currently insufficient in dairy products, these data could be useful for establishing microbiological safety rules for food, including infant formulas. Due to sampling volume and regional limitations, our study could not cover a larger area, but this study can be used as a basis In the future, the research on *Bacillus cereus* prevalence can be carried out on a large scale, such as increasing the number of samples and collection sites and conducting more in-depth research on its pathogenicity.

## Figures and Tables

**Table 1:** Prevalence and contamination level of *B. cereus* in retail infant formulas in Beijing.

Groups	Detection Rate %	Prevailed Distribution of *B. cereus* (MPN/g)				Means of Prevalence (MPN/g)	Range of Prevalence (MPN/g)
		<3	3~10	11~50	>51		
Domestic brands (*n* = 17)	70.6 a	10	2	0	0	1.17	0.36–4.3
Imported brands (*n* = 51)	52.9 a	23	2	2	0	3.52	0.36–46

a, same letter in the same column indicates no significant difference (*p* < 0.05).

**Table 2:** Virulence genes carried by *B. cereus* isolated from infant formulas in Beijing.

Virulence Genes	Detectable Strains	Carrying Rates (%)
*hblA*	5	5.68
*hblC*	8	9.09
*hblD*	5	5.68
*nheA*	48	54.5
*nheB*	56	63.6
*nheC*	74	84.1
*entFM*	19	21.6
*bceT*	19	21.6
*cytK*	19	21.6
*ces*	3	3.41
*hblA + hblC + hblD*	4	4.55
*nheA + nheB + nheC*	47	53.4
*hblA/C/D + nheA/B/C*	4	4.55

**Table 3:** Patterns of virulence genes of *B. cereus* isolated from infant formulas.

Virulence Gene Patterns	*hbla*	*hblc*	*hbld*	*nhea*	*nheb*	*nhec*	*entFM*	*bceT*	*cytK*	*ces*	Carrying Rate (%)	Number of Isolates
I	−	−	−	−	−	−	−	−	−	−	14.77	13
II	−	+	−	−	−	−	−	−	−	−	1.14	1
III	−	−	−	−	−	+	−	−	−	−	13.64	12
IV	−	−	−	−	−	+	+	−	−	−	2.27	1
V	−	−	−	−	−	+	−	+	−	−	3.41	3
VI	−	−	−	−	+	+	−	−	−	−	4.55	4
VII	−	−	−	−	+	+	+	−	−	−	1.14	1
VIII	−	−	−	−	+	+	−	+	−	−	1.14	1
IX	−	−	−	−	+	+	+	+	−	−	1.14	1
X	−	−	−	−	+	+	−	−	+	−	2.27	1
XI	−	−	−	+	−	+	−	−	−	−	1.14	1
XII	−	−	−	+	+	+	−	−	−	−	22.73	20
XIII	−	−	−	+	+	+	+	−	−	−	7.95	7
XIV	−	+	−	+	+	+	−	−	−	−	1.14	1
XV	−	−	−	+	+	+	+	+	−	−	1.14	1
XVI	−	−	−	+	+	+	−	+	−	−	1.14	1
XVII	−	−	−	+	+	+	−	−	+	−	5.68	4
XVIII	−	−	−	+	+	+	+	+	+	−	4.55	4
XIX	−	−	−	+	+	+	−	+	+	−	1.14	1
XX	−	+	−	+	+	+	−	+	+	−	1.14	1
XXI	−	+	+	+	+	+	−	+	+	−	1.14	1
XXII	+	−	−	+	+	+	−	+	+	−	1.14	1
XXIII	+	+	+	+	+	+	−	+	+	−	1.14	1
XXIV	+	+	+	+	+	+	+	+	+	+	3.41	3

Result of samples tested by virulence gene primer pairs: positive +; negative –.

**Table 4:** Antimicrobial drugs susceptibility of *Bacillus cereus* strains isolated from infant formulas in Beijing.

Antimicrobial Drugs	Type	Conc. (μg/disk)	Resistant	Intermediate	Susceptible
GM	Aminoglycosides	10	0	0	88
TET	Tetracycline	30	7	1	80
ERY	Macrolides	15	0	19	69
CHL	Chloramphenicol	30	2	1	85
AMK	Aminoglycosides	30	0	0	88
CIP	Quinolones	5	0	0	88
CC	Lincomycin	2	0	15	73
SXT	Sulfa	1.25/23.75	1	0	87
RIF	Rifampin	5	38	38	12

## Data Availability

The datasets generated for this study are available on request to the corresponding author.
